# Pathological Study on Trigeminal Ganglionitis Among Rabid Dogs in the Philippines

**DOI:** 10.3390/vetsci12040299

**Published:** 2025-03-24

**Authors:** Nuttipa Iamohbhars, Alpha Grace B. Cabic, Boonkanit Markbordee, Ryota Shiina, Natsumi Tamura, Nozomi Shiwa-Sudo, Kazunori Kimitsuki, Mark Joseph M. Espino, Daria Llenaresas Manalo, Satoshi Inoue, Chun-Ho Park

**Affiliations:** 1 Department of Veterinary Pathology, School of Veterinary Medicine, Kitasato University, 23-35-1, Higashi, Towada 034-8628, Aomori, Japan; 2 Research Institute for Tropical Medicine, Department of Health, 9002 Research Drive, Filinvest Corporate City, Alabang, Muntinlupa City 1781, Philippines; 3 Department of Pathology, National Institute of Infectious Diseases, Toyama 1-23-1, Shinjuku-ku, Tokyo 162-8640, Japan; 4 Department of Microbiology, Faculty of Medicine, Oita University, 1-1 Idaigaoka, Hasama-machi, Yufu 879-5593, Oita, Japan; 5 Department of Veterinary Science, National Institute of Infectious Diseases, Toyama 1-23-1, Shinjuku-ku, Tokyo 162-8640, Japan

**Keywords:** histopathology, immunohistochemistry, rabid dog, trigeminal nerve, ganglionitis

## Abstract

The trigeminal nerve is the primary gateway through which the rabies virus enters the brain. Viral infection-related trigeminal neuritis is associated with certain clinical signs. This study investigated trigeminal ganglion histopathology in 92 rabid dogs. Trigeminal ganglionitis was classified into three pathological grades: mild, moderate, and severe. Immunostaining of selected sections was performed using antibodies against lymphocytes (CD3, CD20), stellate cells (glial fibrillary acidic protein, GFAP), macrophages (Iba-1, HLA-DR), ganglion cells (neurofilament, NF), and Schwann cells (S-100) to identify lesion cell types. In moderate and severe cases, double-immunofluorescence staining was performed to determine neuronophagia and Nageotte nodule cell types. Mild (13.0%) cases had minimal morphological changes in ganglion cells; moderate (56.5%) and severe (30.4%) cases showed infected ganglion cells and axons with degenerative necrosis, which were replaced by inflammatory cells. Immunohistochemically, viral antigens were detected in most ganglion cells in mild cases and were significantly reduced in severe cases. The number of CD3-, CD20-, GFAP-, and Iba-1-positive cells increased as the severity progressed, and neuronophagia and Nageotte nodules primarily comprised HLA-DR-positive cells. These findings suggest that the rabies virus reaches the trigeminal ganglion via ascending or descending routes and induces trigeminal neuropathological changes, contributing to neurological symptoms in rabid dogs.

## Simple Summary

Rabies is a zoonotic disease caused by the rabies virus, which is one of the most neurotropic viruses. Brain lesions caused by rabies are characterized by non-suppurative encephalitis and intracytoplasmic inclusions. However, the histopathology of the lesions in the peripheral nervous tissues remains unclear. This study aimed to investigate the histopathology of the trigeminal ganglion in rabid dogs. Ninety-two trigeminal ganglia from rabid dogs were examined. Trigeminal ganglionitis was classified into three grades based on pathological findings: mild (13.0%), moderate (56.5%), and severe (30.4%). The number of inflammatory cell infiltrations, neuronophagia, and Nageotte nodules exhibited a direct correlation with severity. These pathological findings suggest that the rabies virus reaches the trigeminal ganglion via ascending or descending routes and that trigeminal neuropathological changes induced by the rabies virus contribute to neurological symptoms observed in rabid dogs.

## 1. Introduction

Rabies is a fatal neurological, viral, and life-threatening zoonotic disease with worldwide occurrence. It is caused by the rabies virus, a bullet-shaped, single-stranded, enveloped RNA virus belonging to the *Lyssavirus* genus in the *Rhabdoviridae* family, order *Mononegavirales* [1]. The rabies virus is a multi-host pathogen that affects all warm-blooded animals. As a result, the domestic dog represents an important principal reservoir and source of rabies virus infection and transmission to humans [2]. Rabies remains responsible for 40,000–70,000 deaths annually, of whom >40% are children aged <15 years, with most deaths occurring in rural areas of economically disadvantaged and resource-poor regions of Africa and Asia [3]. Up to 99% of human cases are acquired from bites delivered by infected dogs [2,3,4,5]. Rabies exposure results from animal bites and most commonly occurs through skin penetration, delivering the virus into wounds via infected saliva. After a new host is bitten by an infected animal, the rabies virus first enters muscle tissue by binding to nicotinic acetylcholine receptors at the neuromuscular junction [6]. It then spreads through the peripheral nervous system and trans-synaptically between neurons until widespread infection of the central nervous system (CNS) is achieved. The virus also undergoes centrifugal spread from the CNS into peripheral nervous tissues, peripheral non-nervous tissues, and several extra-neural organs [7,8,9,10,11,12]. However, histopathological and immunohistochemical (IHC) studies on the peripheral tissues of rabid animals tend to be less comprehensive than those of the CNS.

During the progression of rabies in dogs, clinical manifestations generally appear in two forms: furious and paralytic [13]. In the furious phase, most cases exhibit altered phonation caused by the paralysis of the laryngeal muscles, dysphagia caused by spasms, and paralysis of the pharyngeal muscles, which leads to excessive salivation or drooling [2]. In the paralytic phase, the most characteristic sign in dogs is a dropped jaw caused by paralysis of the masseter muscles [13]. These clinical manifestations may be associated with trigeminal nerve dysfunction [14]. The trigeminal nerve, the fifth and largest cranial nerve, is composed of three main branches: ophthalmic, maxillary, and mandibular. The ophthalmic and maxillary nerves are purely sensory and innervate areas such as the face, mouth, eyes, and nasal cavity. Only the mandibular nerve contains both motor and sensory components. It provides somatic motor innervation to the mastication muscles, such as the masseter muscles [15,16,17,18]. Thus, it has been concluded that the neurological symptoms observed in rabid dogs (i.e., dropped jaw) are likely caused by dysfunction of the mandibular nerves, resulting in an inability to close the mouth. This leads to an inability to grasp and chew food or drink water properly. In addition, affected animals may be unable to swallow, leading to abundant salivation. However, the direct causes of these clinical signs and the detailed histopathological evidence underlying these symptoms have not been fully elucidated.

To the best of our knowledge, no published studies in literature have examined the histopathological and IHC findings present in trigeminal nerve samples from naturally infected rabid dogs on a large scale. Therefore, this study aimed to describe the histopathological findings and mechanisms of trigeminal ganglionitis in tissues sampled from 92 rabid dogs in the Philippines.

## 2. Materials and Methods

### 2.1. Animals and Clinical Signs

Trigeminal ganglion samples taken from 92 rabid dogs were obtained from the Research Institute for Tropical Medicine (RITM) in the Philippines for post-mortem rabies diagnosis. All brain specimens were diagnosed as positive for the rabies virus antigen using the direct immunofluorescence antibody test (dFAT) at the RITM. Sixty-one of the dogs were discovered after death, while the other thirty-one were euthanized. The 92 dogs (43 males, 34 females, 15 of unknown sex) ranged in age from 2 months to 8 years, with 19 having an unknown age. Fifty-six of the dogs were small in size (<10 kg), thirty-five were middle-sized (10–24 kg), and one was large-sized (≥25 kg). Sixty-one dogs had no history of rabies vaccination, six were vaccinated against the virus, and no information regarding rabies vaccination status was available for the remaining twenty-five. One of the dogs had been confined without household contact, thirty-nine were household pets but roamed outside as well, nine were leashed, nineteen were free-roaming, seventeen were strays, four were confined, and the living conditions were unknown for the remaining three. Most of the dogs exhibited the behavioral changes and clinical symptoms classified as the primary clinical neurological symptoms of canine rabies infection, including unprovoked aggression, aimless running, inexplicable biting of inanimate objects, excess drooling, and limb paralysis. Jaw or tongue paralysis was observed in 76 dogs, while non-specific clinical neurological symptoms, such as restlessness, an apprehensive watchful look, and vomiting were observed in 4. Seven of the dogs exhibited no behavioral changes or clinical symptoms, and five had unknown signs or symptoms.

### 2.2. Histopathological Examination

Bilateral trigeminal ganglia samples from the dogs (n = 92) were fixed in 10% neutral-buffered formalin at room temperature (RT) for at least 72 h. After fixation, the tissues were cleaved, dehydrated with increasing concentrations of ethanol, cleared using Clear Plus (Falma; Tokyo, Japan), and embedded in paraffin. Tissue fragments were cut into 3-μm sections and stained with hematoxylin and eosin (HE), and serial sections were used for IHC analysis. Trigeminal ganglia from 2 vaccinated dogs (a 10-month-old male beagle and a 4-month-old male miniature dachshund) were used as negative controls. Brain sections from other dogs that had been previously confirmed to have rabies via direct immunofluorescence were used as positive controls.

### 2.3. Digitization of Tissue Slides

**Figure 1:**
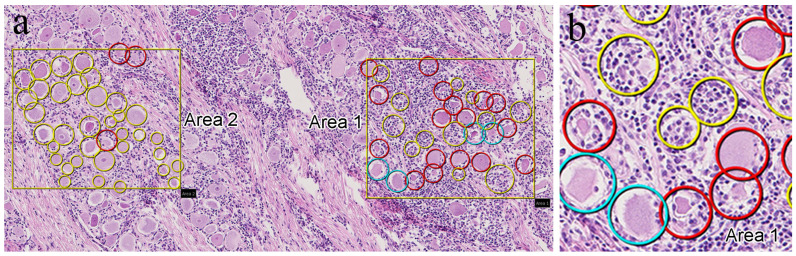
Screenshot images of cell counts: (**a**) low magnification and (**b**) high magnification of area 1. The solid squares (0.2 mm^2^) indicate the cell count areas. At high magnification in (**b**), the yellow circle represents a Nageotte nodule, the red circle represents neuronophagia, and the blue circle represents intact cells. The total number of cells in each area was determined by summing the yellow, red, and blue circles.

To facilitate discussions regarding the histopathological findings among pathologists located in both the Philippines and Japan, all HE slides were scanned in 20× mode using NanoZoomer^®^-SQ (Hamamatsu Photonics, Hamamatsu, Japan), converted into digital images, uploaded to an internet server, and viewed using NZ Connect image-viewing software (NDP viewer 2, Hamamatsu Photonics, Hamamatsu, Japan) for remote pathological analysis. Trigeminal ganglia lesions were evaluated by three trained pathologists and scored based on the degree and presence of inflammatory cell infiltration, neuronophagia, and Nageotte nodules (clusters of satellite cells and macrophages). Trigeminal ganglionitis was scored as mild if scattered inflammatory cell infiltration was present with extremely rare or no evidence of neuronophagia or Nageotte nodules; moderate if there was abundant, multifocal-to-coalescing inflammatory cell infiltration and occasional neuronophagia or Nageotte nodules; and severe if there was abundant inflammatory cell infiltration, frequent neuronophagia, and significant quantities of Nageotte nodules [19]. Additionally, statistical analysis was conducted to determine the proportion of neuronophagia and Nageotte nodules within the total number of ganglion cells. The statistical analysis was performed on two randomly selected 0.2 mm^2^ areas per section from 10 samples, with each area classified as mild, moderate, or severe (Figure 1). Three pathologists independently counted the occurrences of neuronophagia and Nageotte nodules; any disagreements in the results were collaboratively reviewed via Zoom and NZConnect until a consensus was reached.

### 2.4. IHC Analysis

IHC analysis was performed to identify cell types and neurofilament degeneration in the lesions. Except for virus–antigen detection, only selected sections were immunostained with antibodies using the polymer method. To detect viral antigens and cell types in the tissues, we used the following antibodies: rabbit polyclonal rabies phosphoprotein (anti-P) [20], rabbit polyclonal CD3 for T lymphocytes (Agilent Technologies, Santa Clara, CA, USA), rabbit polyclonal CD20 for B lymphocytes (Thermo-Fisher Scientific, Fremont, CA, USA), rabbit polyclonal ionized calcium-binding adaptor molecule 1 (Iba-1; Wako, Japan) and mouse monoclonal HLA-DR (Agilent Technologies) for macrophages, rabbit polyclonal GFAP for satellite cells (Nichirei Bioscience, Tokyo, Japan), mouse monoclonal neurofilament protein (NF) for axons (Agilent Technologies), and mouse monoclonal S-100 protein for Schwann cells (Thermo-Fisher Scientific). Briefly, tissue sections were treated to activate antigens with 0.25% trypsin at RT for 30 min for anti-P antibodies. Microwave treatment was performed at 170 W for 10 min for CD3, CD20, and S-100; for proteinase K at 37 °C for 10 min for NF; and no treatment for GFAP and Iba-1. To remove endogenous peroxidases, tissue sections were treated with 0.3% or 3% hydrogen peroxide (H_2_O_2_) in methanol. To block nonspecific reactions, the sections were treated with 10% normal rabbit or mouse serum. Primary antibodies were diluted 1:1,000 (anti-P), 1:400 (CD20), and 1:500 (Iba-1 and HLA-DR). These dilutions, along with the remaining pre-diluted antibodies (CD3, GFAP, NF, and S-100), were incubated at RT for 30 min or at 4 °C overnight in a humidified chamber. Envision+System-labeled polymer-HRP rabbit antibodies (Agilent Dako) were used to detect CD3, CD20, Iba1, and HLA-DR; Histofine Simple Stain MAX-PO rabbit antibodies (Nichirei Bioscience) were used to detect anti-P and GFAP; and Histofine Simple Stain MAX-PO mouse antibodies (Nichirei Bioscience) were used to detect NF and S-100 (Table 1). Antigen–antibody reactions were visualized using 3,3′-diaminobenzidine (Nichirei Biosciences). The tissue sections were then counterstained with hematoxylin and mounted.

### 2.5. Indirect Immunofluorescence Double-Staining

To identify the antigen-expressing cell types, double-staining of single tissue sections was performed using an immunofluorescent antibody technique. To evaluate the relationship between viral antigen-positive cells and satellite cells, a combination of anti-P antibody and GFAP staining was used, as described in the IHC section. To evaluate the relationship between viral antigen-positive cells and macrophages, a combination of anti-P antibody and HLA-DR staining was used, as described above. To evaluate the relationship between satellite cells and macrophages, a combination of GFAP and HLA-DR staining was employed, as described above. To block non-specific reactions, the sections were treated with 10% normal goat serum. Primary anti-P antibodies and HLA-DR (Agilent Dako) were diluted as described in the IHC section. As secondary antibodies, FITC-conjugate goat anti-rabbit IgG (H+L; Southern Biotechnology Associates, Inc., Birmingham, AL, USA) for anti-P, Alexa Fluor 546-conjugate goat anti-rabbit IgG (H+L) for GFAP (Molecular Probes), and Alexa Fluor 546-conjugate goat anti-mouse IgG (H+L) for HLA-DR (Molecular Probes) were mounted in 1:200 dilutions. DAPI (Thermo-Fisher Scientific) was used for counterstaining.

## 3. Results

### 3.1. Histopathological Findings

Histopathological evaluation showed that the trigeminal ganglia exhibited mononuclear inflammation, neuronophagia, and Nageotte’s nodules at varying intensities between cases. Trigeminal ganglionitis was classified as mild in 12 dogs (13.0%), moderate in 52 (56.5%), and severe in 28 (30.4%).

**Figure 2:**
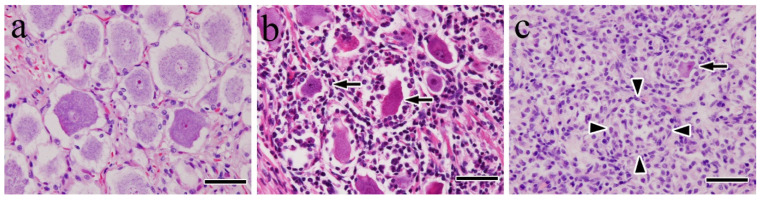
Trigeminal ganglion. Hematoxylin and eosin stain: (**a**) mild, (**b**) moderate, (**c**) severe cases. No histopathological changes were observed in the ganglion cells and axons (**a**). Numerous inflammatory cell infiltrations around the ganglion cells and neuronophagia (arrows) were observed (**b**). Neuronophagia (arrow) and Nageotte nodules (arrowheads) were observed (**c**). Bar = 50 μm.

In the mild cases, histopathological lesions in the trigeminal ganglia consisted of scattered lymphocyte infiltration that rarely formed small, dense, nodular aggregates in the stroma. Morphological changes in the ganglion cells and axonal units were not observed (Figure 2a). In the moderate cases, lymphocyte and macrophage infiltration were high, and significant numbers of ganglion cells exhibited various morphological changes (Figure 2b). The most commonly observed morphological changes in the infected cells were decreased size, pyknotic nuclei, deep eosinophilic cytoplasm, and neuronophagia, although peripheral displacement of the nucleus and central chromatolysis were also occasionally observed.

In severe cases, inflammation was most severe peripherally within the ganglion cells. Infiltrating macrophages abutting ganglion cells were frequently observed, which is consistent with neuronophagia. Nageotte nodules (Figure 2c), characterized by overt neuronal loss with replacement by both satellite cells and infiltrating macrophages, were frequently observed.

Wallerian-like degeneration, including axonal segmental fragmentation, axon swelling (spheroids), vacuolation, and inflammatory cells, such as lymphocytes and macrophages, was apparent in peripheral trigeminal nerve bundles. In all the cases, typical cytoplasmic inclusions (i.e., Negri bodies) were indistinct and could not be identified under high magnification using light microscopy.

The number of neuronophagia and Nageotte nodules in the 10 randomly selected mild, moderate, and severe samples increased as the disease severity progressed. Specifically, the average percentages of neuronophagia and Nageotte nodules were 5.2% and 3.2% in the mild cases, 35.2% and 14.5% in the moderate cases, and 37.1% and 59.2% in the severe cases, respectively. The combined values for neuronophagia and Nageotte nodules were 8.4%, 49.7%, and 96.3% in the mild, moderate, and severe cases, respectively. The majority of the ganglion cells in the severe cases were replaced by neuronophagia or Nageotte nodules.

### 3.2. IHC Findings

**Figure 3:**
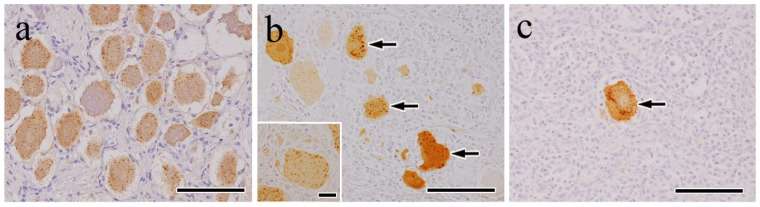
Trigeminal ganglion. Immunohistochemistry for the rabies virus antigen (anti-P): (**a**) mild, (**b**) moderate, and (**c**) severe cases. In mild cases, antigen-positive cells were diffusely observed (**a**), whereas in the moderate ((**b**), arrows) and severe cases ((**c**), arrow), the number of positive cells (arrow) decreased. Viral antigen was found in the cytoplasm of macrophage-like cells in areas of neuronophagia (Figure 2b, top left of the inset). Bar = 100 μm.

Rabies virus antigens were detected in the trigeminal ganglion cells and axons of all of the samples. The antigens appeared as fine granular diffuse patterns in the perikaryons of the ganglion cells and axons. In a few cases, irregularly shaped homogeneous masses were observed in the ganglion cell perikaryons. Notably, the viral antigen-positive ganglion cells were diffusely distributed in the mild cases (Figure 3a). However, in the moderate (Figure 3b) and severe (Figure 3c) cases, viral antigen-positive ganglion cells were present in significantly low numbers or absent entirely from the lesions. Viral antigen positivity was occasionally found in the cytoplasm of satellite-like cells and macrophage-like cells in areas of neuronophagia (Figure 3b, inset) and Nageotte nodules. No viral antigens were detected in the negative control samples.

**Figure 4:**
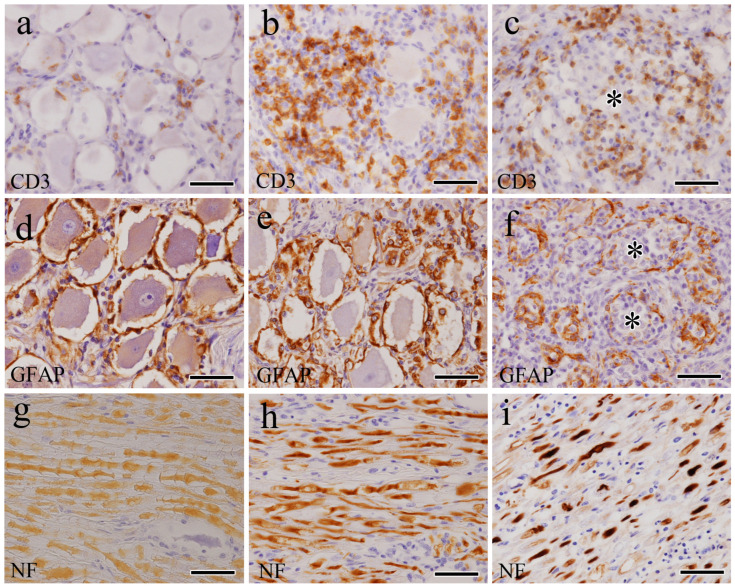
Trigeminal ganglion. Immunohistochemical findings of CD3 (**a**–**c**), GFAP (**d**–**f**), and NF (**g**–**i**) in the trigeminal ganglia of mild (**a**,**d**,**g**), moderate (**b**,**e**,**h**), and severe (**c**,**f**,**i**) cases. As the severity progressed from mild to severe, the number of CD3-positive cells increased; in mild (**a**) and moderate (**b**) cases, such cells were mainly observed in the stroma, whereas in severe (**c**) cases, they were also detected in the Nageotte nodule (asterisk). As the severity progressed from mild (**d**) to severe (**f**), the number of GFAP-positive cells increased; however, in the Nageotte nodule (asterisk) of severe cases, the number of GFAP-positive cells decreased. In mild (**g**) cases, no axonal changes were observed, but in moderate (**h**) and severe (**i**) cases, axonal swelling and fragmentation were observed, along with strong NF positivity. Bar = 50 μm.

As severity progressed from mild to severe, the number of CD3-positive (Figure 4a–c) and CD20-positive cells increased. In mild and moderate cases, CD3-positive and CD20-positive cells were located in the stroma (Figure 4b); in severe cases, many CD3-positive cells were found to infiltrate the Nageotte nodules (Figure 4c).

In mild cases, GFAP-positive cells were detected in a single layer surrounding the ganglion cells (Figure 4d). In moderate and severe cases, the number of positive cells increased, occasionally forming small-sized clusters (Figure 4e). Moreover, the Nageotte nodules of severe cases had fewer GFAP-positive cells (Figure 4f). Iba-1-positive and HLA-DR-positive cells were scattered in the stroma of the mild cases. In moderate and severe cases, there were significantly high numbers of positive cells in the stroma, nerve bundles, and areas of neuronophagia and Nageotte nodules. In axons of mild cases (Figure 4g), there was no change in NF immunoreactivity. However, in moderate and severe cases, strong positivity was observed, particularly in swollen or fragmented axonal segments (Figure 4h,i). No significant changes were observed in the S-100-positive cells.

### 3.3. Indirect Immunofluorescence Double-Stain Findings

**Figure 5:**
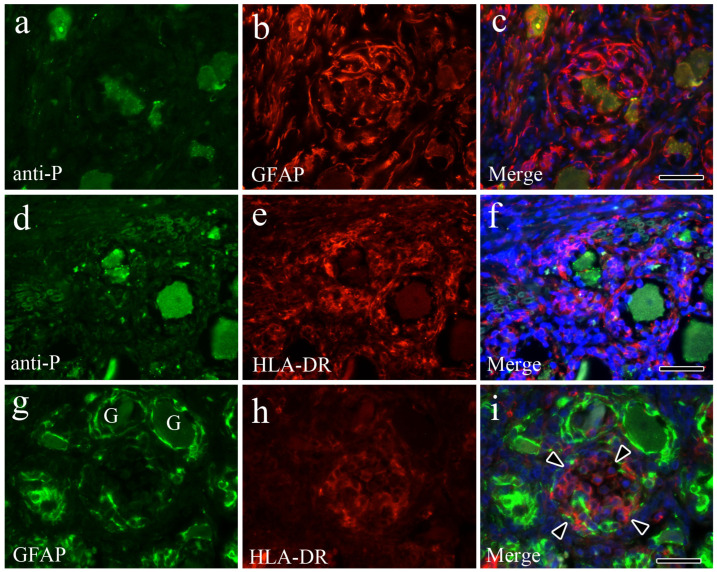
(**a**–**i**) Trigeminal ganglion. Indirect immunofluorescence double-staining. (**a**–**c**) Anti-P and GFAP (moderate cases), (**d**–**f**) anti-P and HLA-DR (moderate cases), and (**g**–**i**) GFAP and HLA-DR (severe cases). In moderate cases, multi-layered GFAP-positive cells surrounded virus-infected ganglion cells (**a**–**c**). In moderate cases, multi-layered HLA-DR-positive cells infiltrated the plasma membranes of virus-infected ganglion cells (**d**–**f**). In severe cases, ganglion cells disappeared, and HLA-DR-positive cells replaced the ganglion cell loss areas (arrowheads, Nageotte nodule) with significant reductions in satellite cells (**g**–**i**). G: ganglion cells. Bar = 50 μm.

Indirect immunofluorescence double-staining was used to investigate mechanisms underlying neuronophagia and Nageotte nodule formation. In moderate cases of trigeminal ganglionitis, double-staining for anti-P and GFAP showed a large number of GFAP-positive cells surrounding the infected ganglion cells (Figure 5a–c). In moderate cases of trigeminal ganglionitis, double-staining for anti-P and HLA-DR in the neuronophagic areas showed a large number of HLA-DR-positive cells infiltrating the plasma membranes of virally infected ganglion cells (Figure 5d–f). In severe cases of Nageotte nodule formation in trigeminal ganglionitis, double-staining for GFAP and HLA-DR revealed that in areas of ganglion cell loss, these cells were replaced by HLA-DR-positive macrophages (Figure 5g–i), with significant reductions in satellite cells. These results suggest that in both neuronophagia and Nageotte nodule formation, virally infected cells were phagocytosed by macrophages, leading to Nageotte nodule formation. These nodules may then coalesce, resulting in significant swelling of the infected ganglion.

## 4. Discussion

This study aimed to describe the pathological features of trigeminal ganglia in rabid dogs. The centrifugal spread of the rabies virus from the CNS to the peripheral nerves along neuronal pathways is essential for transmitting the virus to its natural hosts [21,22,23]. In dogs, the incubation period ranges from 10 days to 6 months. Several factors influence the incubation period, including the age and immune status of the animal; the location, extent, and depth of the wound; and the distance between the wound site and an entry point into the CNS [24]. In this study, trigeminal ganglionitis was observed in trigeminal nerve samples taken from rabid dogs. It was characterized by non-suppurative inflammatory cell infiltration, neuronophagia, and the formation of Nageotte nodules. Inflammation was classified into three categories: mild, moderate, and severe. Most of the cases were classified as moderate, followed by severe and mild. These pathological features correlated directly with the severity of the infection, which depended on the route of viral invasion, duration, and disease stage. Furthermore, naturally infected dogs were discovered post-mortem or were euthanized. Because of this, no information was available regarding infection durations or exact bite locations, making it difficult to clearly correlate pathological stages with disease progression. However, most of the dogs analyzed herein were small-sized, and it was likely that most were aged <1 year. These features facilitated faster viral migration from the bite site to the CNS, with the trigeminal nerve likely representing the first CNS target. This may explain the higher frequency of moderate and severe cases vs. mild ones. If a bite occurs on the face, particularly near the masseter muscle, the rabies virus may use the trigeminal nerve as a direct pathway to the CNS. In such cases, pathological findings in the trigeminal ganglion may show moderate or severe inflammation because of the short time required for the virus to reach the trigeminal nerve. Conversely, if a bite occurs on the tail, leg, or body, the virus may spread through other peripheral nerves to the CNS before reaching the trigeminal nerve. This longer transmission time may result in milder pathological findings in the trigeminal ganglion.

In this study, histopathological examination of trigeminal ganglionitis was conducted using light microscopy and HE staining. Trigeminal ganglionitis was scored based on the degree of inflammation and the presence of inflammatory cell infiltration—particularly mononuclear cells—including lesions of neuronophagia and Nageotte nodules. In the severe cases, most ganglion cells were lost, with Nageotte nodules replacing the vacant areas. These findings are consistent with previous reports of ganglioradiculitis (i.e., sensory neuronopathy) in dogs, which described prominent mononuclear inflammatory cell infiltration, degeneration, and loss of neurons, including axonal degeneration [25,26,27]. In dogs, spontaneous nonsuppurative inflammation of the cranial and spinal ganglia and roots often occurs in conjunction with meningoencephalitis. Ganglioradiculitis is a rare idiopathic disease in dogs, although it can also be associated with certain viral infections, such as rabies, pseudorabies, and canine herpes virus infections [25,27,28]. Moreover, our findings are similar to those reported in sensory ganglia [29,30], the right atrial parasympathetic branch [11], and extraneural brain organs [9] in human rabies cases. These findings are also consistent with previous reports concerning natural rabies cases in animals, such as ganglioneuritis in horses with paralytic rabies [31], and in tissues outside the CNS in bovine rabies [32]. They also align with findings from experimental rabies infections, such as those observed in mice inoculated via peripheral routes [33] and raccoons infected with various urban rabies virus isolates via intramuscular inoculation in the masseter muscle. In raccoons, pathological findings in the trigeminal ganglia have included extensive mononuclear inflammatory infiltration and the presence of Negri bodies within the cytoplasm of ganglion cells [34]. Notably, this differs from our results, wherein Negri bodies were not observed in the trigeminal ganglia of our sample of rabid dogs. Furthermore, our histopathological findings concerning trigeminal ganglionitis in the dogs we analyzed are consistent with those of HIV-induced peripheral neuropathy [35], as well as the trigeminal ganglionitis and dorsal root ganglion damage that have been observed in macaque monkeys infected with the simian immunodeficiency virus [19,36,37].

IHC analysis using an anti-P antibody to detect the rabies virus antigens revealed that they were present in trigeminal ganglion neurons, appearing as several dozen brown granules per cell in a small granular pattern, which is consistent with previous reports. For instance, in natural cases of bovine rabies, rabies virus antigens were detected in the spinal ganglia [32]. In horses, rabies virus antigens were observed in the spinal ganglia of spinal cord gray matter, appearing as small, viral antigen-positive inclusions predominantly located at the cytoplasmic margins of alpha motor neurons [31]. In our study, viral antigen-positive ganglion cells were diffusely distributed in the mild cases, whereas their presence was significantly reduced in the severe ones. This decrease in ganglion cell density may be partially attributed to macrophage infiltration. Morphologically, the infiltrating macrophages replaced lost neurons, indicating overt neuronophagia and the formation of Nageotte nodules [19]. These findings suggest that inflammatory mechanisms were activated, leading to the injury and death of trigeminal ganglion neurons [35], and that cell debris, including virus proteins, was phagocytosed by macrophages as a consequence of degenerative necrosis in infected ganglion cells.

In this study, inflammatory cells mainly comprised CD3-, CD20-, Iba-1-, and HLA-DR-positive cells that infiltrated areas surrounding the trigeminal ganglia and stroma. In mild cases, these cells were detected in lower numbers, with higher numbers being observed in moderate and severe cases. In severe cases, Iba-1- and HLA-DR-positive macrophages were diffusely distributed, particularly in lesions of Nageotte nodules. These findings suggest that a combination of T-cell-mediated cytotoxicity and humoral immune responses contributed to the trigeminal ganglionitis [38]. The activated macrophages exhibited more prominent cellular processes and increased contact with the trigeminal ganglion, suggesting high reactivity and enhanced phagocytic functions. The activation of phagocytic macrophages is likely an attempt to combat the underlying viral infection and clear degenerating neuronal debris [36]. This inflammatory response may play a crucial role in causing damage to and the death of trigeminal ganglion neurons, leading to a loss of inputs to the central sensory pathways [33,35]. This response may also prevent the spread of the rabies virus and act as a defense mechanism to inhibit viral replication, given that rabies virus replication and assembly occur within the neuronal cell body [39,40].

In this study, the mechanisms underlying neuronophagia and Nageotte nodule formation were investigated using indirect immunofluorescence double-staining. Rabies virus antigens were present in the trigeminal ganglion neurons of pathological lesions that exhibited neuronophagia. An increased number of HLA-DR-positive macrophages surrounded and infiltrated the perineuronal compartments of these neurons. In the severe cases, neuronal loss was observed in the lesions with Nageotte nodules, primarily replaced by HLA-DR-positive macrophages and fewer GFAP-positive satellite cells. Satellite cells, which play supportive roles in the peripheral nervous system, are found in the sensory, sympathetic, and parasympathetic ganglia, forming tight, thin sheaths around each neuronal soma [41]. Following nerve injury, satellite cells proliferate in the primary sensory ganglia [42]. Satellite cell proliferation is often observed during the early stages of nerve injury (~3–14 d). After 14 d, these cells increase their expression of genes linked to the MHC II protein complex and leukocyte migration [41]. This finding is consistent with our own, as we observed that the Nageotte nodules present in severe cases of trigeminal ganglionitis were primarily composed of HLA-DR-positive macrophages, with fewer GFAP-positive satellite cells.

When axons in the peripheral nervous system are injured, their distal portions undergo progressive degradation (Wallerian degeneration), which is characterized by the breakdown of both axons and myelin. This complex process is initiated by metabolic or mechanical damage to peripheral nerves, leading to axonal degeneration, myelin breakdown, glial cell proliferation, compromising of the blood–nerve barrier, and the infiltration and activation of macrophages. In addition to the cellular responses elicited by injured axons, Wallerian degeneration involves the dedifferentiation of Schwann cells and the activation of the immune response [43]. In this study, axonal swelling and fragmentation suggesting Wallerian degeneration were observed in severe cases. These degenerated axons showed strong positivity for NF, and Iba-1-positive macrophages infiltrated alongside swollen axons and stroma. These findings are consistent with those of previous reports of ganglioradiculitis in dogs [25,26,27] and closely resemble the findings of reports of natural cases of rabies in humans [29,30], cattle [32], and HIV-induced peripheral neuropathy [35].

Rabies symptoms progress through three phases in dogs. The prodromal phase lasts approximately 2–3 days after symptom onset, during which the animal exhibits behavioral changes before progressing to the furious or to the paralytic stage. Clinical symptoms begin with changes in behavioral patterns. Infected dogs may exhibit altered behaviors, such as apprehension, irritability, or subtle changes in temperament. During the furious phase, dogs become extremely aggressive and display erratic behavior caused by changes in temperament. These can include manic biting and severe agitation. In some cases, death can occur from convulsions without progression to the paralytic stage. The paralytic phase occurs when the excitatory phase is brief or absent. It is characterized by progressive loss of muscle coordination, paralysis, coma, and eventual death. A key sign of this phase is a dropped jaw caused by paralysis of the masseter muscles, which impairs the dog’s ability to swallow and leads to excessive salivation. Paralysis often begins in the affected limb before progressing to the neck and head [2]. Paralysis of the masseter muscles is caused by the bilateral dysfunction of the mandibular branches of the trigeminal nerve, which innervates these muscles. When these branches are affected, the result is an inability to close the mouth, difficulty eating and drinking, and hypersalivation [17,44]. In this study, trigeminal ganglionitis was observed in most rabid dogs. Additionally, as the disease progressed, the loss of ganglion cells and Wallerian degeneration were noted. These findings suggest that trigeminal ganglionitis in rabid dogs is involved in the clinical symptoms observed.

## 5. Conclusions

This study revealed that the rabies virus may reach the trigeminal ganglion by ascending from the cranial trigeminal nerve or descending from the brainstem through the trigeminal nerve fibers. The virus then multiplies in the trigeminal ganglion, leading to progressive trigeminal ganglionitis, accompanied by the activation of the host immune response. Additionally, rabies virus-induced trigeminal neuropathological changes may contribute to neurological symptoms observed in rabid dogs.

## Figures and Tables

**Table 1:** Primary antibodies used for immunohistochemical examination and their respective target cells.

Antibody ^(a)^	Host	Clone	Dilution	Antigen Retrieval ^(b)^	Source	Target Cells
CD3	Rabbit	polyclonal	Prediluted	MW, 170 W, 10 min	Agilent (Dako)	T cells
CD20	Rabbit	polyclonal	1:400	MW, 170 W, 10 min	Thermo	B cells
GFAP	Rabbit	polyclonal	Prediluted	No treatment	Nichirei	Satellite cells
Iba-1	Rabbit	polyclonal	1:500	No treatment	Wako	Macrophages
HLA-DR	Mouse	TAL.1B5	1:500	MW, 170 W, 10 min	Agilent (Dako)	Macrophages
NF	Mouse	2F11	Prediluted	Pro-K, 37 °C, 10 min	Agilent (Dako)	Ganglions, axons
S-100	Mouse	4C4.9	Prediluted	MW, 170 W, 10 min	Thermo	Schwann cells

^(a)^ GFAP: glial fibrillary acidic protein, Iba-1: ionized calcium-binding adaptor molecule 1, NF: neurofilament protein. ^(b)^ MW: microwave, Pro-K: proteinase K.

## Data Availability

The data presented in this study are available upon request from the corresponding author.
